# Actin-Independent Behavior and Membrane Deformation Exhibited by the Four-Transmembrane Protein M6a

**DOI:** 10.1371/journal.pone.0026702

**Published:** 2011-12-05

**Authors:** Yasufumi Sato, Naoki Watanabe, Nanae Fukushima, Sakura Mita, Tatsumi Hirata

**Affiliations:** 1 Division of Brain Function, National Institute of Genetics, Graduate University for Advanced Studies (Sokendai), Mishima, Shizuoka, Japan; 2 Laboratory of Single-Molecule Cell Biology, Tohoku University Graduate School of Life Sciences, Sendai, Miyagi, Japan; 3 Department of Anatomy, Shinshu University School of Medicine, Matsumoto, Nagano, Japan; Emory University, United States of America

## Abstract

M6a is a four-transmembrane protein that is abundantly expressed in the nervous system. Previous studies have shown that over-expression of this protein induces various cellular protrusions, such as neurites, filopodia, and dendritic spines. In this detailed characterization of M6a-induced structures, we found their varied and peculiar characteristics. Notably, the M6a-induced protrusions were mostly devoid of actin filaments or microtubules and exhibited free random vibrating motion. Moreover, when an antibody bound to M6a, the membrane-wrapped protrusions were suddenly disrupted, leading to perturbation of the surrounding membrane dynamics involving phosphoinositide signaling. During single-molecule analysis, M6a exhibited cytoskeleton-independent movement and became selectively entrapped along the cell perimeter in an actin-independent manner. These observations highlight the unusual characteristics of M6a, which may have a significant yet unappreciated role in biological systems.

## Introduction

Due to its unique characteristics, the biological membrane has been viewed as an active component in many cellular processes [1], [2], [3], [4]. One particularly notable characteristic is that of phase separation, which allows the membrane to self-organize into distinct domains containing different biochemical components [5], [6]. One such domain is the cholesterol-rich lipid raft that partitions with many important signaling components and functions as a signaling center [7], [8]. Another notable characteristic of the biological membrane is its intrinsic morphogenetic properties; a simple lipid bilayer can curve inward or outward and elaborate defined shapes, such as tubules, depending on the associated proteins, without the aid of cytoskeletons [9], [10]. These autonomous properties of the biological membrane indicate that it can control many diverse cellular functions.

M6a is a four transmembrane protein concentrated on the membrane edges of neural growth cones [11], [12], [13]. As exemplified by its lipid-like solubility in organic solvents, this protein is extremely hydrophobic and classified as proteolipid or lipophilin [14]. This family includes two highly similar homologues referred to as M6b and proteolipid protein (PLP)/DM20 [13], which are also abundantly expressed in the nervous system [15], [16]. None of the proteolipid/lipophilin family members, however, has been thus far assigned a clear, specific molecular function, while many diverse functions have been postulated in previous studies [17], [18], [19], [20]. Among them, we can acknowledge their potential involvement in membrane dynamics. More specifically, heterologous expression analyses have shown that M6a and M6b affect membrane trafficking and cell surface expression of G protein-coupled receptors such as the μ-opioid receptor and the serotonin transporter [21], [22], [23]. As the most abundant protein in the myelin membrane, PLP/DM20 is synthesized by oligodendrocytes, regulates the regularly-spaced apposition of myelin membranes, and maintains their stability [24], [25], [26]. In doing so, PLP/DM20 interacts with cholesterol and galactosylceramide, a major myelin glycosphingolipid, and partitions into the membrane raft domain [27], [28], thereby achieving a high concentration, along with that of several other proteins, within the intricate sheets of the myelin membranes [29].

Our research has focused on the regulation of cell morphogenesis by M6a. Whereas some previous studies have found that over-expression of M6a in neurons and neuron-like cell lines enhances outgrowth of neurites [30], [31], [32], others have observed that antibody binding to M6a arrests neurite outgrowth in a seemingly gain-of-function manner [33]. Thus, the previous findings do not consistently support either outgrowth promotion or inhibition by M6a, but rather suggest a more complex modifying role for this protein in neuron morphogenesis via as-yet-undefined mechanisms [33]. In the present study, we characterized M6a-induced membrane protrusions. Previously, the cellular protrusions induced by expression of M6a were defined as filopodia or dendritic spines [32], [34], [35], [36]. However, our detailed characterization highlighted various unusual features of the M6a-induced protrusions for these cellular appendages. For example, we observed that most of the M6a-induced structures were simple membrane-wrapped tubules without actin or tubulin-based cytoskeletons and exhibited random vibrating motion. Furthermore, our single-molecule analyses revealed that M6a molecules perform the unique actin-independent behavior of selectively gliding along membrane edges. These cytoskeleton-independent properties of M6a suggest its unique way of regulating cell morphogenesis. Although the cytoskeletons have been postulated as the major mechanical force that structures cell shapes, our results provide a clear example of cytoskeleton-independent cell morphogenesis, which is probably achieved by mutual physical interactions between a membrane protein and membrane lipids.

## Materials and Methods

### cDNA expression constructs

M6a cDNA was isolated as described previously [33]. To create a GFP-fusion construct, the M6a coding sequence was excised and sub-cloned into the pEGFP-C1 vector (Clontech) such that the N-terminal of the M6a protein was fused in-frame with GFP (GFP-M6a). We also constructed M6a protein that was C-terminally tagged with GFP or N-terminally tagged with RFP (RFP-M6a) [37]. All of these M6a constructs exhibited indistinguishable activity in inducing membrane tubules. M6b and DM20 cDNA was amplified by PCR and used for generating M6a/DM-20 chimeric constructs containing the following amino acids: M6a/1-111-DM20/99-242, M6a/1-62-DM20/52-242, M6a/1-26-DM20/16-242, DM20/1-14-M6a/26-62-DM20/52-242, DM20/1-14-M6a/26-278, DM20/1-46-M6a/58-278, and DM20/1-98-M6a/112-278. Because the same amino acids are typically shared between M6a and DM20 at the junctions, the numbering of these amino acids was assigned by assuming the maximal contribution of M6a amino acids to the chimeric proteins. Single amino acid mutants of M6a were generated by a PCR-based site-directed mutagenesis so that the mutations would mimic the natural point mutations identified in the PLP/DM20 gene. The expression constructs were transfected into various cultured cell lines using the lipofection reagent FuGENE (Roche). The construct for the GFP-tagged pleckstrin homology domain from rat PKB was a generous gift from Dr. Kazuo Emoto.

### Primary culture

Hippocampal neurons were prepared from mouse embryos at embryonic day 17. For expression in neurons, GFP-M6a was subcloned in the expression vector pCAGGS [38] and transfected into the dissociated primary neurons using Nucleofector (Amaxa) as described previously [39]. The experiments were performed in strict accordance with the guideline for the Care and Use of Laboratory Animals of the National Institutes of Genetics with every effort to minimize suffering in animals. The protocol was approved by the nimal Committee of the National Institute of Genetics (Permit Number: 23-6).

### Histology

Transfectants were fixed with 4% paraformaldehyde (PFA)/phosphate-buffered saline (PBS) for 30–60 min, immunostained with mAb 1B4 [33] (10 µg/ml) in 10 mM of Tris-HCl (pH 7.4, 130 mM NaCl, 0.1% Tween20 [TBS]) at 4°C overnight, and followed by goat anti-Armenian hamster Ig secondary antibody (Jackson ImmunoReseach). Other antibodies and materials used for staining are as follows: tetramethylrodamine B isothiocyanate (TRITC)-labeled phalloidin (1 µg/ml, Sigma), mouse anti-acetylated tubulin mAb (1∶500, 6-11B-1, Sigma), mouse anti-α-tubulin mAb (1∶500, B-5-1-12, Sigma), rabbit anti-green fluorescent protein (GFP) antibody (1∶1000, MBL), mouse anti-early endosome antigen (EEA)-1 mAb (1∶500, BD Transduction), mouse anti-LAMP-1 mAb (1∶500, H4A3, Santa Cruz). To stain lipid components, the fixed cells were incubated with 1,1′-dioctadecyl-3,3,3′,3′-tetramethylindocarbocyanine perchlorate (DiI-C_18_, 1 µg/ml, Life Technologies), filipin III (10 µg/ml, Sigma), cholera toxin B subunit conjugated with Alexa 488 (10 µg/ml, Life Technologies), or 6-((N-(7-nitrobenz-2-oxa-1,3-diazol-4-yl)amino)hexanoyl)sphingosyl phosphocholine (NBD-C_6_-sphingomyelin, 5 µM, Life Technologies) in PBS at room temperature for 1–3 hours. Conventional electron-microscopy was performed by the methods described by Yonemura et al. [40] with the generous assistance of Ms. Kazumi Shinkura, Dr. Nobuyuki Shiina, and Dr. Makio Tokunaga.

### Time-lapse analyses

Fluorescent images were captured with a charge-coupled device (CCD) camera (Cascade 512B; Photometrics) through water-immersion lenses dipped into a culture dish set in a chamber on an upright microscope (BX61; Olympus) and processed with Slidebook software (Intelligent Imaging Innovations). During imaging, mAb 1B4 (10 µg/ml), latrunculin B (1 µM, Sigma), or methyl-β-cyclodextrin (5 mM, Sigma) was gently dropped into the culture medium to achieve the indicated concentration. Phase-contrast images were obtained with a CCD camera (Sensys; Roper Scientific) on the stage of an inverted microscope (Axiovert 135; Carl Zeiss) and processed with MetaMorph software (Molecular Devices).

### Fluorescent single-molecule analysis

Fluorescent single-molecule microscopy was carried out as previously described [41]. Briefly, *Xenopus* XTC cells were transfected using SuperFect (Qiagen) and spread on a poly-l-lysine-coated glass coverslip attached to a flow cell in 70% Leibovitz's L15 medium (Invitrogen) without serum. The flow cell was placed on the stage of an Olympus BX52 microscope equipped with a cooled CCD camera (Cool SNAP HQ; Roper Scientific) or an Olympus inverted IX71 microscope equipped with UIC-QE (Universal Imaging). Cells expressing low levels of fluorescence-tagged proteins were tracked under illumination of a restricted area with a 75-W xenon illumination system at 21–23°C.

For motion analysis of M6a speckles, the positions of spots were tracked using the Track Object component of MetaMorph software. The tracks were compared with the original images, and only the data that consistently followed the trajectory of a single spot were chosen. The *N* trajectories of the random walk group, *r_i_*(*t*) = [*x_i_*(*t*), *y_i_*(*t*)] (*i* = 1, 2, …, *N*), were analyzed by determining the mean square displacement <*r* ^2^> as a function of time, *t*:
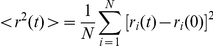
The trajectory data were also analyzed by determining the mean square displacement <*r* ^2^> as a function of the time interval *nδt*. For individual particles, an ensemble average of the square displacement between positions separated by the time interval *nδt* was calculated as:
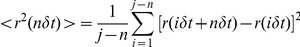
where *j* is the total number of frames [42], [43]. The time intervals were overlapping so that the values of <*r* ^2^(*nδt*)> were not independent, but there was an insufficient number of data points with which to adequately average non-overlapping time intervals. The mean square displacement for the entire random walk group was then calculated by averaging <*r* ^2^(*nδt*)> over the data obtained for multiple M6a speckles.

When particles undergo pure random walk diffusion, the theoretical plots of the mean square displacement versus time are represented by a straight line [42], [44]. For these particles, the diffusion coefficient *D* in two dimensions was defined as:
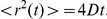



### Quantification of membrane tubules

COS cells were fixed at 2 days after transfection with various cDNA constructs. The number of membrane tubules longer than 5 µm in each transfectant was counted under a fluorescence microscope.

### Adhesion assay

To create stable transfectants, cDNAs for the GFP-fused M6a, M6b, and DM20 were sub-cloned in the pCEP4 vector (Life Technologies). HEK cells were transfected with the cDNA constructs and cultured in the presence of 100 µg/ml of hygromycin B (Roche) for selection. The resulting transfectants and parental cells were dissociated into single cells with 1 mM of EDTA in Ca^2+^/Mg^2+^-free Hank's balanced salt solution containing 20 µg/ml of DNaseI. They were suspended in normal or Ca^2+^/Mg^2+^-free Hank's solution at a concentration of 5×10^5^ cells/ml, and 0.5 ml of the suspension was poured into a well of a 24-well plate (Thermo Scientific) precoated with 10% FBS. After agitation at 80 rpm for 1 hour at 37°C, the number of cells in the resulting aggregate was counted under a microscope. To quantify the extent of cell attachment onto the culture substratum, 5 ml of the cell suspension in DMEM containing 10% FBS was placed on an uncoated 60-mm Petri dish (BD) and stationary incubated in a 5% CO_2_ atmosphere at 37°C for various terms. The dish was then washed with Ca^2+^/Mg^2+^-free Hank's solution twice and the number of the cells attached onto the dish was counted.

## Results

### Deformation of the cell membrane into tubules by M6a

**Figure 1:**
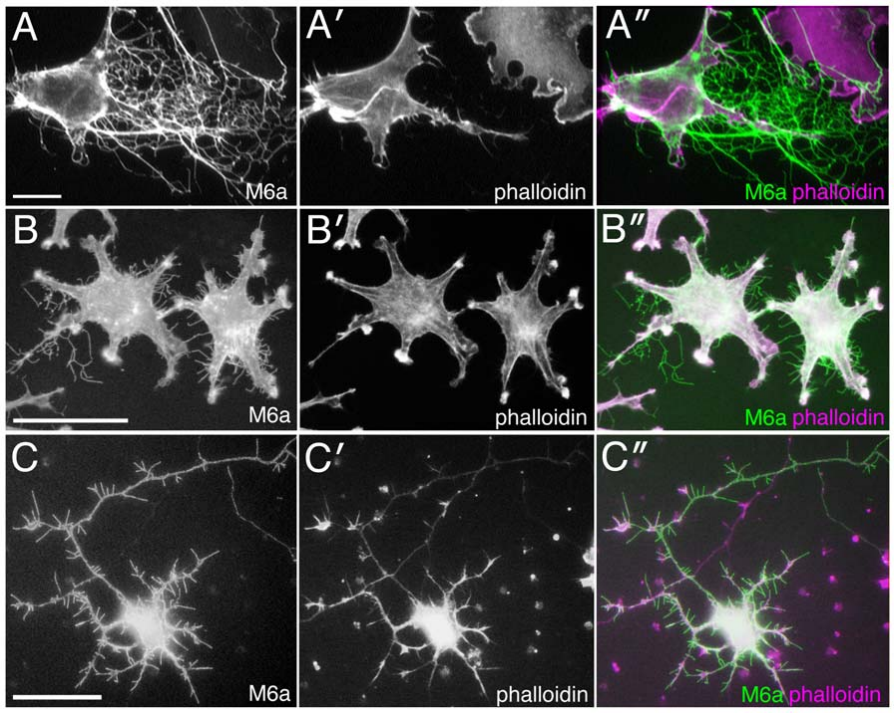
Induction of membrane tubules by expression of M6a. (A) A COS cell transfected with M6a cDNA and doubly stained with mAb 1B4 (A, green in A″) and TRICT-phalloidin (A′, magenta in A″). The membrane tubules are almost imperceptibly labeled with phalloidin. (B) L cells transfected with GFP-M6a (B, green in B″) and stained with TRICT-phalloidin (B′, magenta in B″). (C) A primary cultured hippocampal neuron transfected with GFP-M6a (C, green in C″) and stained with TRICT-phalloidin (C′, magenta in C″). Bars, 50 µm (A—C).

We expressed M6a in various cultured cell lines such as COS [45], CHO [46], neuro2a [47] and L [48] cells, and found drastic deformation of the cell membrane in all the cell lines; long branched membrane protrusions enriched with M6a protein were induced from the rounded cell perimeter (Fig. 1A, B). Many of these protrusions attached to the culture substratum and the surface of other cells, where they branched into and tangled with each other in a complex manner. In primary culture of hippocampal neurons, transfection of M6a also induced a number of membrane protrusions over dendrites, cell bodies and axons (Fig. 1C).

Previous studies had observed a similar induction of protrusions by M6a and defined them as filopodia and dendritic spines [32], [34], [35], [36]. We, however, noticed many unnatural characteristics of the protrusions for these cellular appendages. Notably, phalloidin staining revealed that distal parts of these protrusions did not contain detectable levels of actin fibers (Fig. 1A′—C′, A″—C″), which typically form the structural and motility basis of filopodia and dendritic spines. Moreover, the protrusions did not contain microtubules (data not shown), another major cytoskeleton, further enhancing uncertainty regarding the structural basis of the M6a-induced structure. Many of these protrusions were extremely thin and virtually undetectable under phase-contrast or differential interference-contrast microscopy, but clearly visible under immunolabeling with anti-M6a antibody.

**Figure 2:**
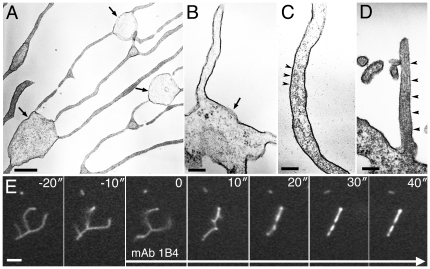
Ultrastructure and movement of membrane tubules induced by M6a. (A) Ultrastructure of the membrane tubules induced in a CHO cell transfected with M6a cDNA. Tubules are trifurcated at the swollen domain (arrows). (B) A high magnification of membrane tubules connected to a swollen branch point (arrow). Cytoskeletal filaments or membrane vesicles are not recognizable. (C) A high magnification of a distally elongated tubule. A few actin-like filaments were barely discernible within the tubule (arrowheads). (D) A short proximal segment of a putative cell filopodium sticking out from the cell body on the bottom. Orderly aligned actin-filaments densely fill the process (arrowheads). (E) Time-lapse imaging of isolated membrane tubules visualized with RFP-M6a at 10-sec intervals. After addition of mAb 1B4 at 0 sec, the tubules abruptly freeze. Bars, 500 nm (A), 200 nm (B—D), and 2 µm (E).

Electron microscopy analysis confirmed the unusual characteristics of these ramified protrusions; they were simple membrane tubules of approximately 100–200 nm in width and wrapped in lipid bilayers (Fig. 2A). The branching points often swelled up spherically and contained few electron-dense materials (Fig. 2A, B). Both of the spherical branches and tubular parts scarcely contained membrane vesicles or cytoskeletal filaments. Although a few isolated actin-like filaments were occasionally encountered along the distally elongated membrane tubules (Fig. 2C), the content of these filaments was remarkably lower, compared with that in short proximal protrusions, putative normal filopodia, which stuck straight up from the cell body and were packed with tightly bundled actin filaments (Fig. 2D). The low content of actin filaments in the distal protrusions is probably the reason for the undetectable labeling with phalloidin (Fig. 1). Because the physiological counterpart of the M6a-induced thin protrusions remain unknown, they are hereafter referred to as membrane tubules.

### Dynamic movement of M6a-induced membrane tubules

The findings thus far raise an important question: If the M6a-induced membrane tubules do not contain cytoskeletons, how do they form? When M6a was fused with a fluorescent protein, we were able to observe the formation of membrane tubules in live imaging. Many of the tubules actually formed as retraction fibers when the cell edge dragged during migration (Movie S1). Specifically, the cell movement pulled the cell membrane between the withdrawing edge and the anchor on the substratum and elongated it as a tubule.

Observation of the dynamics of the membrane tubules allowed for identification of another distinguishing feature: An enormous number of membrane tubules were detached from and fell off from the cell bodies, subsequently scattering around the M6a-transfected cells. Surprisingly, these fragments stably maintained their tubular shape and exhibited rapid trembling motion in culture medium for hours (Movie S2, Fig. 2E). Because they were now only cellular debris, and thus could not be defined as living organisms, their movement appeared to be free random motion devoid of expenditure of cellular metabolic energy. In fact, even after fixation with paraformaldehyde, the biologically inactivated tubules still displayed random rapid movement in an energy-free salt solution (data not shown). Interestingly, many fragments had free ends, indicating that maintaining tension is not requisite to maintaining an elongated tubular structure (Movie S2).

As would be expected from their actin-deficient nature, these fragmented tubules were resistant to disruption of actin filaments by treatment with latrunculin B (Movie S3). They maintained free dynamic movement, although this treatment rapidly shrank the cell bodies and cellular filopodia, indicating that the actin fibers were clearly not the structural basis of these tubules. On the other hand, when anti-M6a monoclonal antibody (mAb) 1B4 [33] was added to the tubules, they shrank and consolidated in a dramatic manner and halted their trembling motion within tens of seconds (Figure 2E, Movie S4), indicating that M6a protein is indeed the foundation that shapes the flexible tubular structure.

### Potential interactions of M6a with membrane lipids

**Figure 3:**
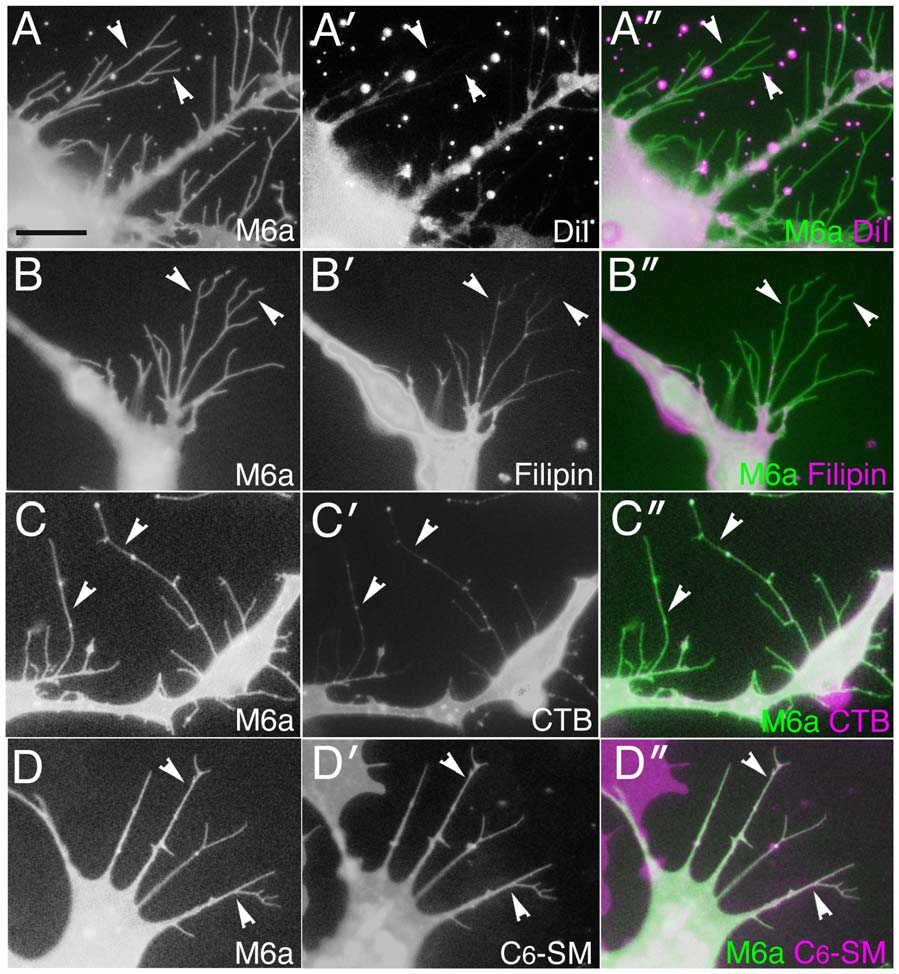
Labeling of membrane tubules with lipid probes. (A—D) Transfectants expressing fluorescent protein-tagged M6a are labeled with DiI-C_18_ (A), filipin (B), cholera toxin B subunit (C), or NBD-C_6_-sphingomyelin (D). The images of M6a are shown in white in the left columns (A—D) and green in the right columns (A″—D″). The images stained with the lipid probes are shown in white in the middle columns (A′—D′) and magenta in the right columns (A″—D″). Membrane tubules (arrowheads) are not fully filled with DiI-C_18_, filipin, or cholera toxin B (A—C). Bar, 10 µm (A—D).

Another unique feature of the M6a-induced membrane tubules observed in this study is that they were not effectively filled with a lipophilic membrane tracer, DiI-C_18_ (Fig. 3A). This tracer selectively partitioned to either the liquid-disordered non-raft or liquid-ordered raft domain, depending on experimental conditions [49], [50], [51]. To determine whether selective partitioning of lipid raft components underlay this unusual membrane morphogenesis, the tubules were stained with several lipid probes. Two raft markers—filipin, which binds cholesterol, and the cholera toxin B subunit that binds ganglioside GM1—exhibited very similar staining patterns, only faintly labeling the tubules and often leaving their distal tips unlabeled (Fig. 3B, C). Although a non-raft marker, NBD-C_6_-sphingomyelin [50], more fully filled the membrane tubules up to the distal ends, the labeling was not heavily concentrated in the membrane tubules (Fig. 3D). Thus, the observations do not strongly support selective partitioning of lipid raft component in or out the membrane tubules. In many systems, methyl-β-cyclodextrin is used to deplete cholesterol and thereby to disrupt the raft domain [50], [52]. However, the treatment with 10 mM methyl-β-cyclodextrin for 30 min neither changed the labeling patterns with the lipid probes (data not shown) nor affected structure and movement of the membrane tubules (Movie S5), refuting the possibility that the membrane lipid raft is the structural basis of membrane tubules. These findings, considered together, do not strongly support the contribution of lipid rafts to membrane tubulation, but do suggest a somewhat distinct specialization of tubular membrane that leads to the poor labeling with DiI-C_18_.

**Figure 4:**
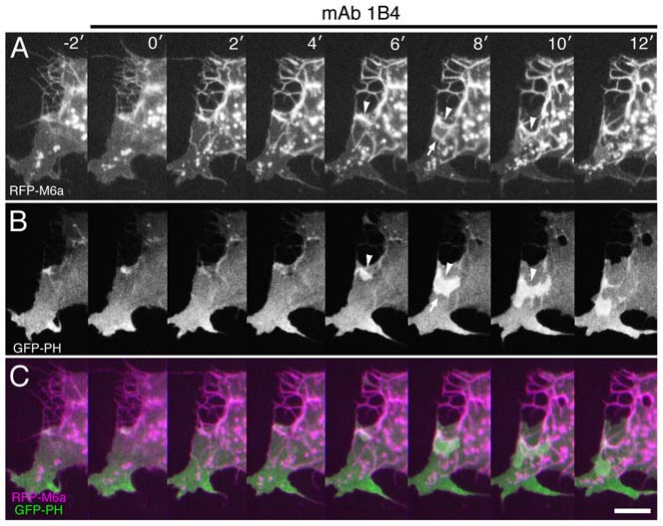
Activation of membrane ruffling upon addition of anti-M6a mAb 1B4. Time-lapse images of a COS cell co-transfected with RFP-M6a (A, magenta in C) and GFP-PH (B, green in C) taken at 2-min intervals. After mAb 1B4 is added at 0 min, RFP-M6a is aggregated and the M6a-aggregated membrane gradually withdraws (arrowheads). At the same time, membrane ruffles labeled with GFP-PH ignite and spread while in contact with the aggregates (arrows). Bar, 10 µm.

In live cell imaging, membrane ruffling is readily visualized with a GFP-tagged pleckstrin homology domain (GFP-PH) [53], which binds to phosphatidylinositol trisphosphate (PIP3), an important lipid-mediated signaling mediator [4], [54]. In parental COS cells, the membrane ruffling marked with PIP3 signals always started locally on the cell perimeter, and then laterally propagated along the perimeter (Movie S6). In M6a transfectants, the membrane ruffling was detectable only rarely in the cell perimeter and never in the membrane tubules (Fig. 4), consistent with the observation that the roundish transfectants were mostly deficient of membrane ruffling. However, when the transfectants were treated with anti-M6a mAb 1B4, the ruffling membrane associated with intense PIP3 signals bursted not only from the perimeter but also dorsally and centrally throughout the cell body in an uncoordinated manner (Fig. 4, Movie S7). As had that of the fragmented membrane tubules (Fig. 2E, Movie S4), the mAb treatment instantaneously consolidated the membrane tubules over the cell bodies, with the resulting aggregates gradually receding in tandem with withdrawal of the cell perimeter (Fig. 4). Thus, the enhanced uncoordinated production of membrane ruffling could be a compensatory reaction performed by the cells to counteract the shrinkage and squeezing of the cell edge. The mAb-aggregated membrane appeared to be an obstacle to the smooth propagation of membrane ruffling, because it always abruptly terminated by the aggregates (Fig. 4).

### Widespread involvement of amino acids spanning the first transmembrane domain in membrane tubulation

**Figure 5:**
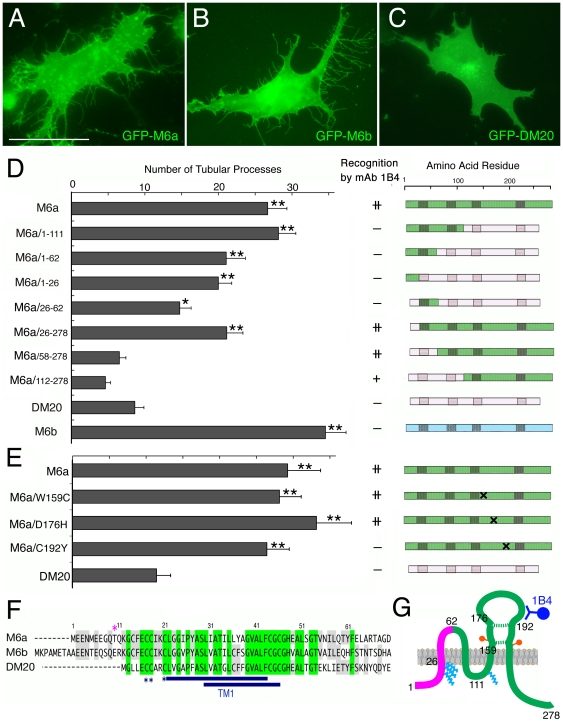
Membrane tubulation activities of M6a and its derivatives. (A–C) Fluorescent images of COS cells expressing GFP-M6a (A), GFP-M6b (B), and GFP-DM20 (C). M6a and M6b, but not DM20, induce membrane tubules. Bar, 50 µm. (D, E) Membrane tubulation activities and structures of the proteolipid family members, M6a/DM20 chimeric proteins (D), and M6a single- point mutants (E). The gray bars display the average numbers of membrane tubules (± SEM) per cell (*n* = 30 for each). *P<0.002, **P<0.0002 by Student's t-testing compared with the DM20 transfectants. The binding of mAb 1B4 to each protein is marked in the middle column. The diagrams on the right side display the structures of individual proteins; the green, pink, and blue regions indicate the amino acid stretches derived from M6a, DM20, and M6b, respectively. The shaded areas indicate the putative transmembrane domains and the cross indicates the position of a mutation. (F) Alignment of the N-terminal sequences of M6a, M6b, and DM20. The amino acids common to all three and any two members are shaded in green and gray, respectively. The two blue underlined regions indicate the transmembrane domains predicted by two different algorithms: SMART (upper) and Swiss-Prot (lower). The blue asterisks indicate the sites for acylation with fatty acids determined for DM20 [65] and the magenta asterisk indicates a potential protein kinase C phosphorylation site. (G) Structure and function of M6a. Sites for internal disulfide bonds (dotted green lines), acylation with fatty acids (light blue _zigzags), and glycosylation (orange circles) were empirically determined from the reported structure of PLP/DM20 [57], [65]. The N-terminal domain encompassing the first transmembrane domain (magenta) is responsible for the membrane tubulation. The mAb 1B4 (blue) recognizes the second extracellular loop. The amino acid landmarks for mutant constructions are numbered.

The three members of the proteolipid family, M6a, M6b, and PLP/DM20, are highly homologous over their entire length [13]. DM20 is a splicing isoform of PLP with a 35-amino acid deletion that is more similar to M6a than PLP [55]. When expressed in cultured cells, M6b exhibited a slightly stronger membrane-tubulation activity than did M6a (Fig. 5A, B, D), while DM20 did not induce production of membrane tubules in COS cells (Fig. 5C, D). These results indicate the existence of clear differences in the potency of tubule induction among the proteolipid family members, which is likely attributable to the differences in their amino acid sequences.

The proteolipid family lacks a cleavable signal sequence for insertion into the cell membrane [56], [57], and simple truncations or alterations of the internal amino acids easily result in mutant proteins being stacked in the endoplasmic reticulum [58], [59]. We, therefore, constructed M6a/DM20 chimeric proteins by carefully swapping the homologous sequences and examined induction of membrane tubules by these proteins (Fig. 5D). Through screening of various chimeric proteins, the activity to induce tubules was mapped onto the N-terminal half of the M6a protein (M6a/1-111), whereas the C-terminal half did not exhibit significant activity (M6a/112-278). Subdivisions of the M6a N-terminal sequence decreased the tubulation activity, but we consistently detected the activity in the chimeric protein containing the N-terminal 62 amino acids (M6a/1-62). When this domain was further divided into two parts, both halves exhibited weak tubulation activity when complemented with the remainder of the DM20 sequences (M6a/1-26, M6a/26-62), although the effects were not always statistically significant. These results suggest that membrane tubulation is not achieved by any single small domain within the M6a protein but rather involves a concerted effort among the M6a-specific amino acids that scatter throughout the N-terminal sequence that spans the first transmembrane domain (Fig. 5F, G).

As mAb 1B4 recognizes M6a but neither M6b nor DM20, using the M6a-DM20 chimeric proteins, we mapped the epitope recognized by mAb 1B4. Because the mAb recognizes M6a in live cells, the epitope must be exposed extracellularly. The mAb 1B4 labeled the chimeric protein in which only the second extracellular domain was derived from M6a (M6a/112-278) but not the protein in which only the first extracellular domain was derived from M6a (M6a/1-111), indicating that the epitope is positioned in the second extracellular loop (Fig. 5D). We made several single amino acid substitutions in the second extracellular loop of M6a, and found that mAb 1B4 did not bind the protein of which cysteine 192 was altered to tyrosine (M6a/C192Y) (Fig. 5E). This homologous cysteine in DM20 engages in disulfide bonding with another cysteine in the same second extracellular domain (Fig. 5G). Thus, the disulfide-bonded structure between the conserved cysteines in M6a may be required for the recognition by mAb 1B4. This point mutation at cysteine 192, however, did not change the membrane tubulation activity (Fig. 5E). Therefore, although mAb 1B4 profoundly disrupted the membrane tubules (Figure 2E, Movie S4), the positions of the epitope and the membrane tubulation activity were separable molecularly.

### Dynamic entrapment of M6a protein on membrane edges in a cytoskeleton-independent manner

**Figure 6:**
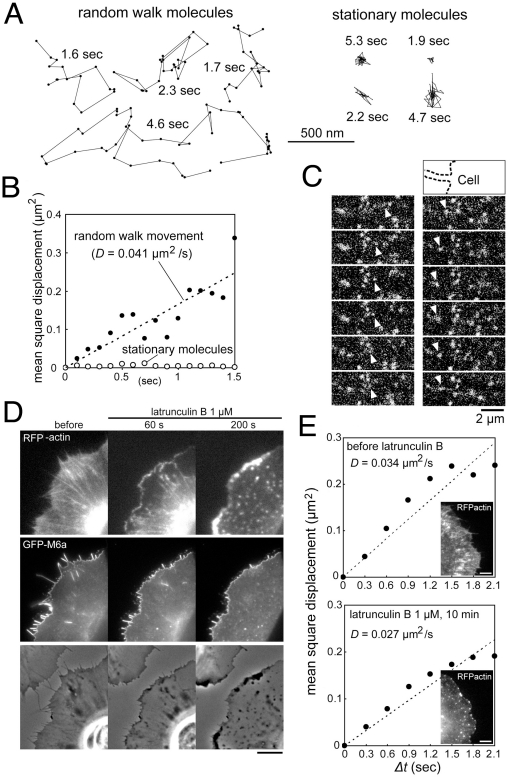
Actin-independent movement of M6a molecules. (A) Representative trajectories of GFP-M6a speckles acquired at 100-ms intervals in XTC cells. The fluorescence intensities of individual spots are comparable to those of single GFP-actin molecules [41], suggesting that each spot contains one or a few GFP-M6a molecules. The behavior of the fluorescent spots can be categorized into two groups, designated the random walk (left) and stationary (right) groups. The duration of each trajectory is indicated in seconds. (B) Mean square displacements of the M6a speckles in the random walk (closed circles *n* = 27) and stationary (open circles *n* = 17) groups. The plot for the random walk group can be represented by a linear line (dashed), indicating that M6a largely undergoes random diffusion with a diffusion coefficient of 0.041 µm^2^/sec. (C) Examples of GFP-M6a speckles (arrowheads) exhibiting random diffusive motion toward the cell edge (left) and along a filopodium (right) shown at 100-ms intervals. The upper drawing indicates the cell contour (dashed line). (D) A cell coexpressing RFP-actin (top panels) and GFP-M6a (middle panels) treated with 1 µM of latrunculin B. The bottom panels display the differential interference-contrast images. Although most of the actin-rich filopodia retract after treatment, the M6a proteins remain associated with the edge. Note the strong actin depositions near the cell edge at 200 sec that are accompanied by almost no M6a molecules. Bar, 5 µm. (E) Mean square displacements of M6a speckles in the random walk group before and at 10 min after treatment with 1 µM of latrunculin B. Each plot is approximated by a linear line (dashed), yielding similar diffusion coefficients regardless of the extent of actin cytoskeleton disruption. The insets display RFP-actin at the indicated time points. Bars, 5 µm.

The unique morphogenetic activity of M6a motivated examination of its molecular behavior. GFP-M6a was expressed at a low level in thin XTC cells, and movement of single or oligomeric M6a molecules was tracked in fluorescent microscopy [41]. Owing to the relatively low time resolution, this analysis could not evaluate the influence of microscale membrane compartments [43], but did reveal the overall random walk diffusion of fluorescent molecules over a two-dimensional membrane plane (Fig. 6A, B, Movie S8). When they reached the cell perimeter, the molecules were abruptly trapped on the edge, where they turned to engage in one-dimensionally restricted movement (Fig. 6C, Movie S8). In the filopodia, both back and forth gliding motions were obvious. The molecules then translocated from the filopodia to the lamellipodial edge and vice versa as if there were a unified one-dimensional track along the entire cell perimeter.

Dual-color imaging of the molecules with actins or tubulins (Movie S9) revealed that most M6a molecules did not follow these cytoskeletal cables. Furthermore, depolymerization of actin filaments by addition of 1 µM of latrunculin B did not significantly alter the movement of M6a molecules and the diffusion coefficients roughly estimated from the movement (Fig. 6D, E, Movie S10). This treatment rapidly destroyed the actin networks in the filopodia and lamellipodia, leading to their withdrawal. However, even on the withdrawing cell edge, M6a molecules were persistently entrapped (Fig. 6D, Movie S10). This observation clearly contrasts with previous reports describing other membrane edge proteins as translocating only to the advancing edge along the actin cytoskeleton and rapidly disassembling from the withdrawing edge [60], [61], [62], and also significantly differs from the previously reported M6a behavior examined with antibody-coated beads [63] (see Discussion).

### Enhancement of adhesive properties in M6a and M6b transfectants

**Figure 7:**
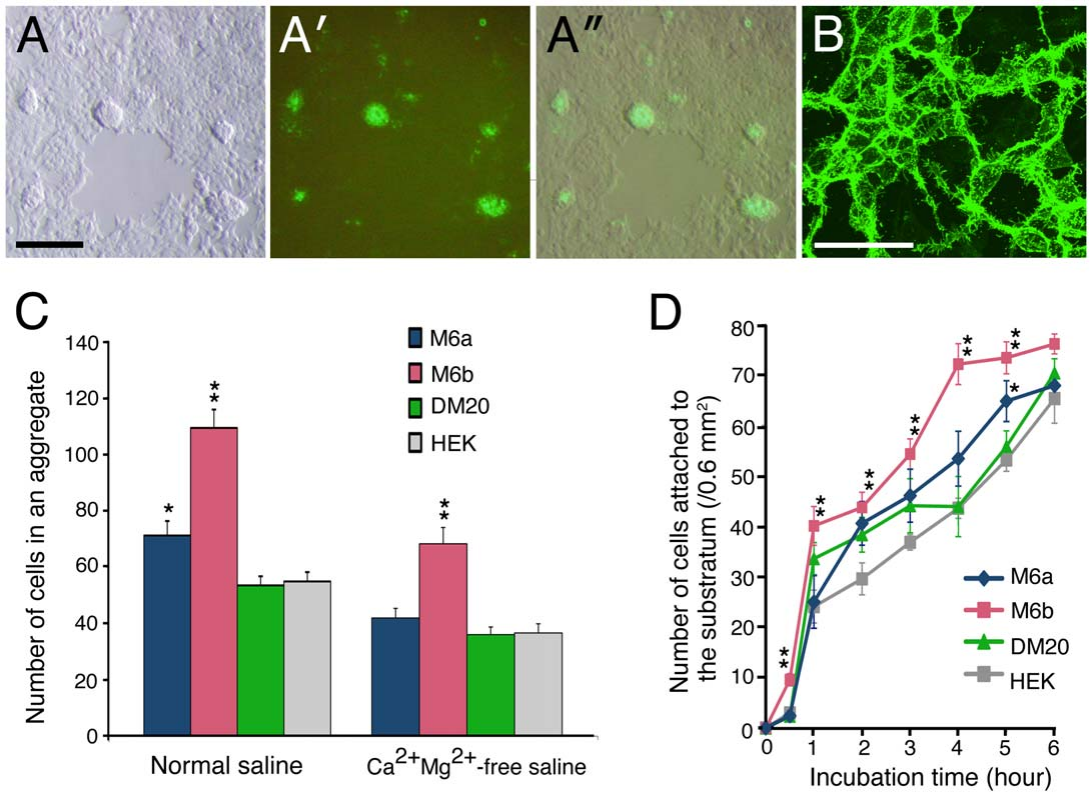
Adhesive properties of M6 transfectants. (A) HEK cells stably expressing GFP-M6a in differential interference contrast (A), fluorescent (A′), and the merged (A″) views. The cells display a strong tendency to clump together during long-term culturing. (B) Confocal fluorescence image of the M6a transfectants. Bars, 100 µm (A), 50 µm (B). (C) The average number of cells (± SEM) in an aggregate formed under agitation culturing with normal or Ca^2+^/Mg^2+^-free saline (*n* = 10 for each). *P<0.05, **P<0.001 by t-testing compared with the parental HEK cells. (D) Average number of cells (± SEM) that have attached to the substratum within the indicated time period (*n* = 6 for each). *P<0.05, **P<0.001 by t-testing compared with the parental HEK cells.

We constructed HEK cell lines that stably express M6a protein, although the expression was not very constant and easily lost in a fraction of the cells during passages. These M6a transfectants revealed a strong tendency to clump together in a monolayer culture (Fig. 7A—A″). The piled-up cells always strongly expressed M6a, whereas the cells in the flat monolayer entirely lost or substantially reduced their M6a expression. Although the M6a protein appeared abundant around the boundaries of the cells, closer observation revealed its distribution was not precisely junctional on the cell–cell contact plane, as is that of a typical cell-adhesion protein, but rather concentrated on thin processes intertwining between the cells (Fig. 7B).

Because the observation of cell clumping suggested that the M6a transfectants had higher adhesive properties, cell-aggregation assay was performed using the stable transfectants. Regardless of the presence or absence of divalent ions, the M6a transfectants consistently formed a slightly larger aggregate compared with parental HEK cells over independently repeated experiments, although the difference was only statistically significant in the assay with normal saline containing Ca^2+^ and Mg^2+^ ions (Fig. 7C). The M6b transfectants, which also revealed a similar clumping phenotype in culture, exhibited a stronger adhesiveness and one that reached a higher level of statistical significance, whereas the DM20 transfectants exhibited the same adhesive activity as parental HEK cells (Fig. 7C). The enhancement of adhesiveness was not mediated by homophilic interactions between M6 molecules, because the transfectants also enhanced the adhesiveness to parental HEK cells (data not shown). Furthermore, the transfectants increased adhesiveness to uncoated Petri dishes in the same relative magnitude measured in the cell aggregation assay (Fig. 7D), indicating that the transfectants increase nonspecific adhesion properties to various substrata.

### Enlargement of early endosomes during mAb 1B4-induced endocytosis

**Figure 8:**
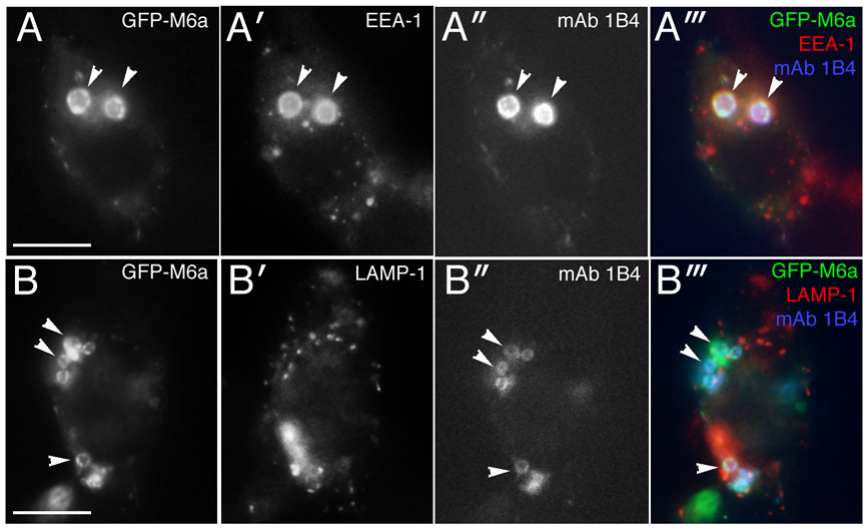
Enlargement of endosomes in M6a transfectants treated with mAb 1B4. (A—B) HEK cells at 2 hours after addition of mAb 1B4 (10 µg/ml). Images for GFP-M6a (A, B, green in A‴ and B‴), an early endosome marker, EEA-1 (A′, red in A‴), an late endosome marker, LAMP-1 (B′, red in B‴), and endocytosed mAb 1B4 (A″, B″, blue in A‴ and B‴). Bars, 10 µm.

M6a has been associated with membrane trafficking [21], [23]. When the HEK M6a stable transfectants were treated with anti-M6a mAb 1B4, we noticed rapid enlargement of intracellular membrane compartments (Movie S11). Because binding of antibodies to membrane proteins typically induce endocytosis of the antibody-bound proteins, we suspected that the enlarged membrane compartments might be the mAb 1B4-induced endosomes. Immunostaining for endosome markers indeed revealed that the enlarged compartments are the early endosomes that had incorporated the mAb-bound M6a (Fig. 8A). The late endosomes were seemingly unaffected by the mAb 1B4 treatment and did not contain internalized M6a protein (Fig. 8B). Thus, the internalized mAb-bound M6a appeared to disturb the endocytic pathway at the level of early endosome, leading to accumulation and fusion of early endosomes in the M6a transfectants.

## Discussion

Previous studies have reported induction of various cellular protrusions by expression of M6a [30], [31], [32], [34], [35], [36]. Several of the reported structures closely resemble the membrane tubules that we describe here, indicating that we observed similar phenomena. Nevertheless, there are several potential discrepancies between previous and our observations. For example, the membrane lipid raft was critical for the M6a-induced formation of filopodia [35], whereas we did not find that it significantly contributed to the formation of membrane tubules. Likewise, a previous report concluded that cysteine192 was essential for filopodial induction [34], whereas we did not find it to be very important in our membrane tubulation assay. There also exist discrepancies among the findings regarding the inducibility of protrusions by DM20 [36]. One reason for such discrepancies may be that previous studies sought to identify common ground between the M6a-induced and physiological cellular structures, and therefore limited the analysis to specimens that bore a greater degree of biological characteristics, e.g., cytoskeleton-containing structures. In contrast, we simply examined M6a-induced structures, regardless of physiological relevance. In fact, the more we examined the membrane tubules, the more difficulty we experienced in relating them to a well-documented physiological structure. The cytoskeleton-independent processes have not been well appreciated in the field of cell morphogenesis. We hope that our finding that a membrane protein can shape a subcellular structure without the aid of cytoskeletons will call for more attention to the potential of this novel morphogenetic force and lead to future discoveries of biological structures or phenomena that are more directly related to those observed in the present study.

Recent studies have challenged the hypothesis that the cell membrane is simply a passive structure organized by the underlying cytoskeleton [1], [2], [3]; intracellular endocytic proteins with a curved membrane-binding domain can physically distort the membrane and invaginate the membrane to form tubules. The morphological features of these tubules, such as cytoskeletal independency, are very similar to those induced by over-expression of M6a protein. Because curvature generation has been theoretically predicted for membrane proteins [1], it is possible that M6a generates outward membrane curvature when the proteins are abundantly accumulated. Consistent with this idea, the membrane tubulation activity of M6a was mapped onto the membrane-spanning domain. Interestingly, a previous study reported that PLP deforms liposomes when reconstituted into them [64]. Thus, the proteolipid family members might inherently possess the membrane deforming activity, although PLP was not as potent as M6a or M6b in our membrane tubulation assay. It is important to note that membrane-tubulating activity is associated with curvature-sensing ability, because the curved membrane-binding domain prefers to bind to a suitably curved membrane rather than a flat membrane [1]. We assume that curvature sensing is one of the few mechanisms that can explain the actin-independent recruitment of molecules on the membrane edges, because the highly curved edge membrane could provide a non-cytoskeletal one-dimensional track that stretches along the entire cell perimeter encompassing filopodial and lamellipodial edges.

Previously, Sheetz et al. [63] happened to chase the M6a molecules in growth cones using mAb-immobilized beads [12] without knowing the molecular identity, and reported a similar restricted one-dimensional diffusion along the growth cone edge. The authors assumed that the controlling force arose from the actin cytoskeleton, and attributed the sustained recruitment of M6a on the edge after cytochalasin D treatment to interactions with residual actin filaments. Our findings, however, indicate that M6a molecules continue the same movement after disruption of actin filaments without exhibiting affinity for the residual actin deposits, suggesting independency between their behavior and that of the actin cytoskeleton. The discrepancy between the previous and our findings may be due to cell-type specific mechanisms or technical differences, among which the mAb-assisted method used in the previous study may be especially responsible [63]. The recent finding showed that anti-M6a mAbs trigger a gain-of-function effect and dramatically affect distribution of M6a molecules [33]. Thus, the mAb-assisted method might have influenced the movement on the M6a molecules.

The present study showed that over-expression of M6a or M6b enhances cell adhesiveness and induces cell-cell clumping. Because the transfectants increased general, nonspecific adhesion properties to various substrata, this enhanced adhesiveness may not be directly mediated by M6 molecules, but can involve an indirect consequence of M6a/M6b overexpression. For example, the long membrane tubules induced by the overexpression could greatly expand the contact area with other cells or culture dishes and eventually enhance the attachment to these substrata. It is also possible that the membrane trafficking was affected by the overexpression and altered expression profiles of cell surface molecules including cell adhesion and extracellular matrix attachment proteins. Although there is no direct evidence that membrane trafficking is affected in the M6a/M6b transfectants, we in fact observed that anti-M6a mAb 1B4 severely disturbed the endocytic pathway at the level of early endosomes in the M6a transfectants. Further studies will be needed to address these possibilities and explain the phenotypic alterations in the M6a/M6b transfectants.

Binding of mAb 1B4 to endogenous M6a in axons induces growth-cone arrest in a gain-of-function manner [33]. In the present study, we observed that the same mAb drastically disrupts M6a-induced membrane tubules and prominently enhances the subsequent membrane ruffling. Therefore, it is interesting to relate the mAb-induced growth cone arrest and the mAb-affected membrane dynamics observed in this study. However, their connections have not been so far supported by our unpublished observations. First, another anti-M6a mAb, M6, inhibits axon outgrowth more strongly than mAb 1B4 [33] but does not disrupt membrane tubules in our assays. Second, the M6a-DM20 chimeric protein (M6a/58-278) that does not induce membrane tubules, can mediate axon outgrowth arrest when bound by anti-M6a mAbs. Thus, our current data favor the hypothesis that M6a-induced membrane tubulation is an activity that can be separated from that of mAb-induced axon-outgrowth arrest and has an independently significance in biological processes.

## Supporting Information

Movie S1 **Formation of retraction fibers during migration of an M6a transfectant.** Fluorescence image of a COS cell transfected with GFP-M6a cDNA captured at 1-min intervals.
(MOV)

Movie S2 **Free movement of M6a-induced membrane tubules.** Fluorescence images of tubular fragments enriched with RFP-M6a captured at 10-sec intervals. The tubules display rapid random motion.
(MOV)

Movie S3 **Resistance of M6a-induced membrane tubules to latrunculin B.** Fluorescence images of tubular fragments enriched with RFP-M6a captured at 10-sec intervals. Latrunculin B (1 µM) is present during the period marked “LatB” in the upper left corner.
(MOV)

Movie S4 **Disruption of M6a-induced membrane tubules by mAb 1B4.** Fluorescence images of tubular fragments enriched with RFP-M6a captured at 10-sec intervals. The tubules exhibiting rapid trembling motion are suddenly disrupted by the addition of mAb 1B4, which is present during the period marked “mAb 1B4” in the upper left corner.
(MOV)

Movie S5 **Resistance of M6a-induced membrane tubules to methyl-β-cyclodextrin.** Fluorescence images of tubular fragments enriched with RFP-M6a captured at 10-sec intervals. Methyl-β-cyclodextrin (10 mM) is present during the period marked “MCD” in the upper right corner.
(MOV)

Movie S6 **Normal membrane ruffling in a COS cell.** Fluorescence images of a COS cell transfected with GFP-PH alone.
(MOV)

Movie S7 **Effect of mAb 1B4 on membrane dynamics of an M6a transfectant.** Fluorescence images of a COS cell transfected with GFP-PH (middle) and RFP-M6a (left) cDNA. The merged images are shown in the right panel. The mAb is present during the period marked “mAb 1B4” in the upper right corner.
(MOV)

Movie S8 **Movement of M6a molecules.** Fluorescent imaging of GFP-M6a single molecules taken at 200-ms intervals. Note the random diffusion in the cell body and the one-dimensionally restricted movement along the edge. Bar, 5 µm.
(MOV)

Movie S9 **Double-color imaging of M6a-GFP and cytoskeletal components.** (Left) Double-color imaging of GFP-M6a (green) and actin-RFP (red) at 10-sec intervals. (Right) Double-color imaging of GFP-M6a (green) and tubulin-RFP (red) at 20-sec intervals. Bar 5 µm.
(MOV)

Movie S10 **Effect of latrunculin B on movement of M6a molecules.** Fluorescent speckle imaging of GFP-M6a molecules taken at 300-ms intervals before (left) and at 10 min after (right) application of 1 µM of latrunculin B. Although the treatment destroys the actin-based filopodia seen in the left panel, the drug does not significantly affect the movement of M6a molecules. Bar, 5 µm.
(MOV)

Movie S11 **Enlargement of endosomes in M6 transfectants after addition of mAb 1B4.** Phase-contrast images of HEK M6a transfectants captured at 1-min intervals. The mAb 1B4 is present during the period marked “mAb 1B4” in the upper left corner.
(MOV)
